# 5-Chloro-3-[(*E*)-1,2-diphenyl­ethen­yl]-1*H*-indole

**DOI:** 10.1107/S1600536810034719

**Published:** 2010-09-08

**Authors:** M. NizamMohideen, G. Bhaskar, P. T. Perumal

**Affiliations:** a Department of Physics, The New College (Autonomous), Chennai 600 014, India; b Organic Chemistry Division, Central Leather Research Institute, Chennai 600 020, India

## Abstract

In the title compound, C_22_H_16_ClN, the pyrrole system makes a dihedral angle of 68.9 (1)° with the plane of phenyl ring at the ethenyl 1-position. An intra­molecular C—H⋯π inter­action is observed. In the crystal, inter­molecular C—H⋯π inter­actions link the mol­ecules into infinite chains running along the *b* axis.

## Related literature

For the synthesis and potential uses of indole derivatives, see: Bhuvaneswari *et al.* (2007 ▶); Ghosh & Maiti (2007 ▶); Sakai *et al.* (2008 ▶); Kakiuchi & Kochi (2008 ▶). For the general synthetic procedure and structure analysis of a derivative of the title compound, see: Bhaskar *et al.* (2010 ▶). For standard bond lengths, see Allen *et al.* (1987 ▶).
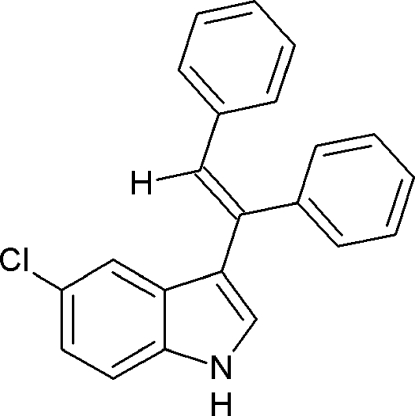


## Experimental

### 

#### Crystal data

C_22_H_16_ClN
*M* *_r_* = 329.81
Monoclinic, 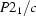

*a* = 10.8869 (6) Å
*b* = 14.0373 (8) Å
*c* = 10.7978 (4) Å
β = 91.706 (2)°
*V* = 1649.42 (14) Å^3^
*Z* = 4
Mo *K*α radiation
μ = 0.23 mm^−1^
*T* = 298 K
0.35 × 0.22 × 0.20 mm

#### Data collection

Bruker Kappa APEXII CCD diffractometer
Absorption correction: multi-scan (*SADABS*; Bruker, 2004 ▶) *T*_min_ = 0.923, *T*_max_ = 0.955
12914 measured reflections
3928 independent reflections
2804 reflections with *I* > 2σ(*I*)
*R*_int_ = 0.023

#### Refinement

*R*[*F* ^2^ > 2σ(*F* ^2^)] = 0.041
*wR*(*F* ^2^) = 0.108
*S* = 1.32
3928 reflections
221 parameters
H atoms treated by a mixture of independent and constrained refinement
Δρ_max_ = 0.24 e Å^−3^
Δρ_min_ = −0.23 e Å^−3^

### 

Data collection: *APEX2* (Bruker, 2004 ▶); cell refinement: *APEX2* and *SAINT* (Bruker, 2004 ▶); data reduction: *SAINT* and *XPREP* (Bruker, 2004 ▶); program(s) used to solve structure: *SHELXS97* (Sheldrick, 2008 ▶); program(s) used to refine structure: *SHELXL97* (Sheldrick, 2008 ▶); molecular graphics: *ORTEP-3* (Farrugia, 1997 ▶); software used to prepare material for publication: *SHELXL97* and *PLATON* (Spek, 2009 ▶).

## Supplementary Material

Crystal structure: contains datablocks global, I. DOI: 10.1107/S1600536810034719/im2219sup1.cif

Structure factors: contains datablocks I. DOI: 10.1107/S1600536810034719/im2219Isup2.hkl

Additional supplementary materials:  crystallographic information; 3D view; checkCIF report

## Figures and Tables

**Table 1:** Hydrogen-bond geometry (Å, °) *Cg*1and *Cg*2 are the centroids of the C10–C15 and C3–C8 rings, respectively.

*D*—H⋯*A*	*D*—H	H⋯*A*	*D*⋯*A*	*D*—H⋯*A*
C18—H18⋯*Cg*1	0.93	2.74	3.573 (2)	150
C20—H20⋯*Cg*2^i^	0.93	2.97	3.690 (2)	136

Symmetry code: (i) 
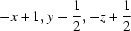
.

**Fig. 1.**
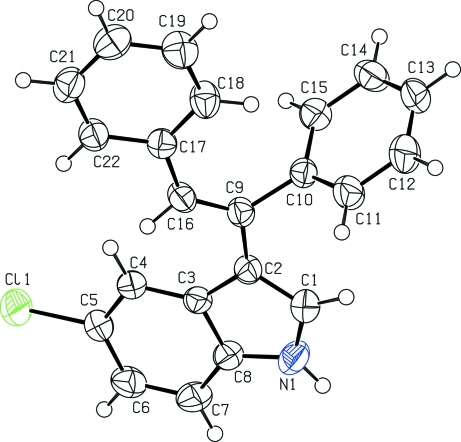
Molecular structure of the title compound with the atom numbering scheme. Displacement ellipsoids are drawn at the 50% probability level. H atoms are presented as small spheres of arbitrary radius.

**Fig. 2.**
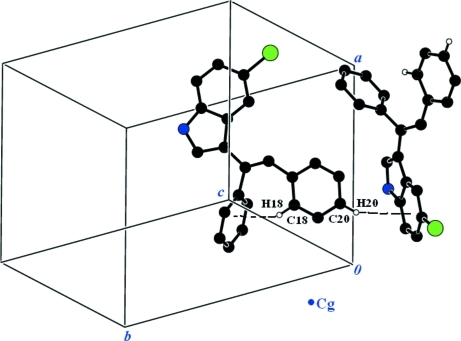
C—H···π interactions (dashed lines) in the title compound. Cg denotes the ring centroid. [Symmetry codes: (i) x, y, z; (ii) 1 - x, -1/2 + y, 1/2 - z]

## supplementary crystallographic information

### Comment

Bhuvaneswari and co-workers (2007) investigated the reaction of indoles with alkynyl alcohols employing platinum as catalyst. Indium(III) bromide is known to catalyze intramolecular cyclization of 2-alkynylanilines (Sakai *et al.*, 2008). The unique properties of indium halides and indium trifluoromethanesulfonate such as non-toxicity, stability in air, and water tolerance has also been described (Ghosh & Maiti 2007). Development of methodologies for heteroarene functionalization *via* C—H activation provides useful applications such as fluorescent dyes, synthetic analogues of natural products, and pharmaceuticals (Kakiuchi & Kochi, 2008). Against this background the structure of the title compound was determined by *X*–ray diffraction.

A perspective view of the title compound with the atom-numbering scheme is shown in Fig. 1. In the structure, all bond lengths and angles are within normal ranges (Allen *et al.*, 1987). The chlorine atom deviates from the least squares planes of the C3—C8 benzene ring by 0.040 (1) Å. The dihedral angle between the pyrroline ring and phenyl ring (C10—C15) is 68.9 (1) °. The indole ring is planar as expected, the maximum deviation from the least squares plane being 0.034 (1) Å for atom C5. The dihedral angle between the phenyl rings C17—C22 and C24—C29 is 78.5 (2) °.

In the crystal structure two C—H···π interactions are observed. One intramolecular interaction determines the conformation of the molecules whereas one intermolecular interaction links the molecules to infinite chains. The C—H···π interactions involve rings C18—H18···*Cg*1^i^ (separation of 2.74 Å) and C20—H20···*Cg*2^ii^ (separation of 2.97 Å, Table 1, *Cg*1 is the centroid of the C10—C15 ring and *Cg*2 is the centroid of the C3—C8 ring).

### Experimental

A mixture of diphenylacetylene (2.4 mmol), 5-Chloro indole (2 mmol) and indium tribromide (0.2 mmol) in toluene (4 ml) was stirred at 383 K for the appropriate time. After completion of the reaction as indicated by TLC, the reaction mixture was diluted with water and extracted with ethyl acetate. The combined organic layers were dried over anhydrous Na_2_SO_4_, concentrated in vacuo and purified by column chromatography on silica gel (Merck, 100 - 200 mesh) to afford the desired product after crystallization (yield: 86%).

### Refinement

All H atoms were positioned geometrically, with C—H = 0.93 Å and N—H = 0.84 Å. H atoms bonded to carbon were constrained to ride on their parent atoms, with *U*_iso_(H) = *xU*_eq_(C), where *x* = 1.5 for methyl H and *x* = 1.2 for all other H atoms. H1N was refined freely.

### Crystal data

**Table:**

C_22_H_16_ClN	*F*(000) = 688
*M**_r_* = 329.81	*D*_x_ = 1.328 Mg m^−^^3^
Monoclinic, *P*2_1_/*c*	Mo *K*α radiation, λ = 0.71073 Å
Hall symbol: -P 2ybc	Cell parameters from 3737 reflections
*a* = 10.8869 (6) Å	θ = 2.4–27.7°
*b* = 14.0373 (8) Å	µ = 0.23 mm^−^^1^
*c* = 10.7978 (4) Å	*T* = 298 K
β = 91.706 (2)°	Block, colourless
*V* = 1649.42 (14) Å^3^	0.35 × 0.22 × 0.20 mm
*Z* = 4	

### Data collection

**Table:**

Bruker Kappa APEXII CCD diffractometer	3928 independent reflections
Radiation source: fine-focus sealed tube	2804 reflections with *I* > 2σ(*I*)
graphite	*R*_int_ = 0.023
ω and φ scan	θ_max_ = 28.3°, θ_min_ = 2.4°
Absorption correction: multi-scan (*SADABS*; Bruker, 2004)	*h* = −14→10
*T*_min_ = 0.923, *T*_max_ = 0.955	*k* = −18→18
12914 measured reflections	*l* = −14→10

### Refinement

**Table:**

Refinement on *F*^2^	Primary atom site location: structure-invariant direct methods
Least-squares matrix: full	Secondary atom site location: difference Fourier map
*R*[*F*^2^ > 2σ(*F*^2^)] = 0.041	Hydrogen site location: inferred from neighbouring sites
*wR*(*F*^2^) = 0.108	H atoms treated by a mixture of independent and constrained refinement
*S* = 1.32	*w* = 1/[σ^2^(*F*_o_^2^) + (0.0422*P*)^2^] where *P* = (*F*_o_^2^ + 2*F*_c_^2^)/3
3928 reflections	(Δ/σ)_max_ = 0.001
221 parameters	Δρ_max_ = 0.24 e Å^−^^3^
0 restraints	Δρ_min_ = −0.23 e Å^−^^3^

### Special details

**Table:**

Geometry. All e.s.d.'s (except the e.s.d. in the dihedral angle between two l.s. planes) are estimated using the full covariance matrix. The cell e.s.d.'s are taken into account individually in the estimation of e.s.d.'s in distances, angles and torsion angles; correlations between e.s.d.'s in cell parameters are only used when they are defined by crystal symmetry. An approximate (isotropic) treatment of cell e.s.d.'s is used for estimating e.s.d.'s involving l.s. planes.
Refinement. Refinement of *F*^2^ against ALL reflections. The weighted *R*-factor *wR* and goodness of fit *S* are based on *F*^2^, conventional *R*-factors *R* are based on *F*, with *F* set to zero for negative *F*^2^. The threshold expression of *F*^2^ > σ(*F*^2^) is used only for calculating *R*-factors(gt) *etc*. and is not relevant to the choice of reflections for refinement. *R*-factors based on *F*^2^ are statistically about twice as large as those based on *F*, and *R*- factors based on ALL data will be even larger.

### Fractional atomic coordinates and isotropic or equivalent isotropic displacement parameters (Å^2^)

**Table:**

	*x*	*y*	*z*	*U*_iso_*/*U*_eq_	
C1	0.50474 (15)	0.38298 (11)	0.55429 (14)	0.0442 (4)	
H1	0.4308	0.4161	0.5542	0.053*	
C2	0.52390 (13)	0.29966 (10)	0.49125 (12)	0.0353 (3)	
C3	0.65154 (13)	0.27519 (10)	0.51474 (12)	0.0339 (3)	
C4	0.72997 (13)	0.20455 (10)	0.47216 (13)	0.0381 (3)	
H4	0.7013	0.1582	0.4169	0.046*	
C5	0.85081 (13)	0.20491 (11)	0.51365 (13)	0.0417 (4)	
C6	0.89718 (14)	0.27230 (12)	0.59749 (14)	0.0493 (4)	
H6	0.9785	0.2686	0.6261	0.059*	
C7	0.82271 (15)	0.34403 (12)	0.63776 (14)	0.0494 (4)	
H7	0.8528	0.3903	0.6923	0.059*	
C8	0.70140 (14)	0.34575 (11)	0.59495 (13)	0.0398 (4)	
C9	0.43048 (12)	0.24722 (10)	0.41772 (12)	0.0353 (3)	
C10	0.32774 (13)	0.30607 (10)	0.36239 (13)	0.0353 (3)	
C11	0.22693 (15)	0.32999 (12)	0.42986 (14)	0.0508 (4)	
H11	0.2238	0.3121	0.5127	0.061*	
C12	0.13000 (16)	0.38050 (12)	0.37533 (16)	0.0580 (5)	
H12	0.0624	0.3964	0.4217	0.070*	
C13	0.13352 (15)	0.40699 (12)	0.25378 (16)	0.0524 (4)	
H13	0.0676	0.4397	0.2171	0.063*	
C14	0.23442 (15)	0.38539 (11)	0.18569 (15)	0.0502 (4)	
H14	0.2377	0.4046	0.1034	0.060*	
C15	0.33150 (13)	0.33484 (11)	0.23991 (13)	0.0417 (4)	
H15	0.3996	0.3202	0.1936	0.050*	
C16	0.43655 (13)	0.15227 (11)	0.40398 (12)	0.0393 (3)	
H16	0.5036	0.1243	0.4451	0.047*	
C17	0.35713 (13)	0.08460 (10)	0.33620 (12)	0.0358 (3)	
C18	0.23999 (14)	0.10354 (11)	0.28396 (15)	0.0467 (4)	
H18	0.2056	0.1637	0.2930	0.056*	
C19	0.17550 (15)	0.03436 (12)	0.21967 (15)	0.0532 (4)	
H19	0.0985	0.0488	0.1851	0.064*	
C20	0.22244 (15)	−0.05556 (13)	0.20551 (15)	0.0524 (4)	
H20	0.1780	−0.1015	0.1612	0.063*	
C21	0.33554 (16)	−0.07694 (12)	0.25734 (14)	0.0494 (4)	
H21	0.3679	−0.1378	0.2491	0.059*	
C22	0.40145 (14)	−0.00790 (11)	0.32190 (13)	0.0425 (4)	
H22	0.4778	−0.0236	0.3570	0.051*	
Cl1	0.95028 (4)	0.11701 (3)	0.46169 (4)	0.06518 (18)	
N1	0.60946 (13)	0.41032 (10)	0.61706 (13)	0.0498 (4)	
H1N	0.6191 (16)	0.4607 (14)	0.6598 (16)	0.070 (6)*	

### Atomic displacement parameters (Å^2^)

**Table:**

	*U*^11^	*U*^22^	*U*^33^	*U*^12^	*U*^13^	*U*^23^
C1	0.0442 (9)	0.0412 (9)	0.0470 (9)	0.0070 (7)	0.0014 (7)	−0.0023 (7)
C2	0.0394 (8)	0.0331 (8)	0.0335 (8)	0.0034 (6)	0.0016 (6)	0.0022 (6)
C3	0.0386 (8)	0.0327 (7)	0.0303 (7)	−0.0002 (6)	−0.0013 (6)	0.0011 (6)
C4	0.0404 (8)	0.0362 (8)	0.0374 (8)	0.0014 (6)	−0.0048 (6)	−0.0033 (6)
C5	0.0386 (8)	0.0430 (9)	0.0431 (9)	0.0072 (7)	−0.0028 (6)	−0.0003 (7)
C6	0.0415 (9)	0.0549 (10)	0.0506 (9)	−0.0018 (8)	−0.0122 (7)	−0.0020 (8)
C7	0.0532 (10)	0.0476 (9)	0.0468 (9)	−0.0069 (8)	−0.0106 (7)	−0.0097 (8)
C8	0.0463 (9)	0.0365 (8)	0.0365 (8)	0.0006 (7)	−0.0013 (7)	−0.0024 (6)
C9	0.0328 (7)	0.0362 (8)	0.0368 (8)	0.0045 (6)	0.0015 (6)	0.0011 (6)
C10	0.0348 (7)	0.0313 (7)	0.0396 (8)	0.0016 (6)	−0.0016 (6)	−0.0022 (6)
C11	0.0505 (10)	0.0595 (11)	0.0427 (9)	0.0174 (8)	0.0055 (7)	0.0036 (8)
C12	0.0458 (10)	0.0673 (12)	0.0613 (12)	0.0218 (9)	0.0066 (8)	−0.0043 (9)
C13	0.0469 (10)	0.0491 (10)	0.0604 (11)	0.0137 (8)	−0.0133 (8)	−0.0027 (8)
C14	0.0548 (10)	0.0509 (10)	0.0442 (9)	0.0024 (8)	−0.0096 (8)	0.0077 (7)
C15	0.0376 (8)	0.0445 (9)	0.0431 (9)	−0.0006 (7)	0.0023 (7)	0.0012 (7)
C16	0.0365 (8)	0.0395 (8)	0.0417 (8)	0.0057 (7)	−0.0051 (6)	0.0043 (7)
C17	0.0374 (8)	0.0351 (8)	0.0351 (8)	−0.0011 (6)	0.0040 (6)	0.0035 (6)
C18	0.0363 (8)	0.0399 (9)	0.0637 (10)	0.0012 (7)	−0.0003 (7)	−0.0007 (7)
C19	0.0377 (9)	0.0542 (11)	0.0673 (11)	−0.0060 (8)	−0.0053 (8)	−0.0009 (9)
C20	0.0521 (10)	0.0496 (10)	0.0556 (10)	−0.0110 (8)	0.0011 (8)	−0.0076 (8)
C21	0.0581 (10)	0.0382 (9)	0.0522 (10)	0.0015 (8)	0.0045 (8)	−0.0049 (7)
C22	0.0444 (9)	0.0399 (9)	0.0430 (8)	0.0046 (7)	0.0010 (7)	0.0031 (7)
Cl1	0.0498 (3)	0.0732 (3)	0.0715 (3)	0.0262 (2)	−0.0158 (2)	−0.0199 (2)
N1	0.0554 (9)	0.0405 (8)	0.0536 (8)	0.0029 (7)	−0.0008 (7)	−0.0156 (7)

### Geometric parameters (Å, °)

**Table:**

C1—N1	1.364 (2)	C12—C13	1.366 (2)
C1—C2	1.3723 (19)	C12—H12	0.9300
C1—H1	0.9300	C13—C14	1.374 (2)
C2—C3	1.4464 (19)	C13—H13	0.9300
C2—C9	1.4692 (19)	C14—C15	1.388 (2)
C3—C4	1.3955 (19)	C14—H14	0.9300
C3—C8	1.4134 (19)	C15—H15	0.9300
C4—C5	1.3770 (19)	C16—C17	1.4655 (19)
C4—H4	0.9300	C16—H16	0.9300
C5—C6	1.394 (2)	C17—C22	1.3954 (19)
C5—Cl1	1.7454 (15)	C17—C18	1.4044 (19)
C6—C7	1.371 (2)	C18—C19	1.374 (2)
C6—H6	0.9300	C18—H18	0.9300
C7—C8	1.386 (2)	C19—C20	1.372 (2)
C7—H7	0.9300	C19—H19	0.9300
C8—N1	1.376 (2)	C20—C21	1.371 (2)
C9—C16	1.343 (2)	C20—H20	0.9300
C9—C10	1.5003 (18)	C21—C22	1.382 (2)
C10—C11	1.3768 (19)	C21—H21	0.9300
C10—C15	1.3845 (19)	C22—H22	0.9300
C11—C12	1.388 (2)	N1—H1N	0.849 (19)
C11—H11	0.9300		
			
N1—C1—C2	110.43 (14)	C11—C12—H12	119.9
N1—C1—H1	124.8	C12—C13—C14	119.99 (15)
C2—C1—H1	124.8	C12—C13—H13	120.0
C1—C2—C3	105.95 (13)	C14—C13—H13	120.0
C1—C2—C9	125.59 (13)	C13—C14—C15	119.88 (15)
C3—C2—C9	128.44 (12)	C13—C14—H14	120.1
C4—C3—C8	118.18 (13)	C15—C14—H14	120.1
C4—C3—C2	134.83 (12)	C10—C15—C14	120.54 (14)
C8—C3—C2	106.89 (12)	C10—C15—H15	119.7
C5—C4—C3	118.57 (13)	C14—C15—H15	119.7
C5—C4—H4	120.7	C9—C16—C17	131.93 (13)
C3—C4—H4	120.7	C9—C16—H16	114.0
C4—C5—C6	122.54 (14)	C17—C16—H16	114.0
C4—C5—Cl1	119.25 (11)	C22—C17—C18	116.39 (13)
C6—C5—Cl1	118.20 (12)	C22—C17—C16	117.23 (13)
C7—C6—C5	119.88 (14)	C18—C17—C16	126.38 (13)
C7—C6—H6	120.1	C19—C18—C17	120.89 (15)
C5—C6—H6	120.1	C19—C18—H18	119.6
C6—C7—C8	118.25 (14)	C17—C18—H18	119.6
C6—C7—H7	120.9	C20—C19—C18	121.33 (15)
C8—C7—H7	120.9	C20—C19—H19	119.3
N1—C8—C7	130.09 (14)	C18—C19—H19	119.3
N1—C8—C3	107.44 (13)	C21—C20—C19	119.28 (15)
C7—C8—C3	122.46 (14)	C21—C20—H20	120.4
C16—C9—C2	121.43 (13)	C19—C20—H20	120.4
C16—C9—C10	122.76 (13)	C20—C21—C22	119.93 (15)
C2—C9—C10	115.80 (12)	C20—C21—H21	120.0
C11—C10—C15	118.76 (13)	C22—C21—H21	120.0
C11—C10—C9	121.34 (13)	C21—C22—C17	122.16 (14)
C15—C10—C9	119.87 (12)	C21—C22—H22	118.9
C10—C11—C12	120.53 (15)	C17—C22—H22	118.9
C10—C11—H11	119.7	C1—N1—C8	109.26 (13)
C12—C11—H11	119.7	C1—N1—H1N	126.3 (12)
C13—C12—C11	120.27 (15)	C8—N1—H1N	124.3 (12)
C13—C12—H12	119.9		
			
N1—C1—C2—C3	−1.52 (17)	C16—C9—C10—C15	−83.04 (18)
N1—C1—C2—C9	177.44 (13)	C2—C9—C10—C15	98.36 (15)
C1—C2—C3—C4	−174.63 (16)	C15—C10—C11—C12	1.1 (2)
C9—C2—C3—C4	6.5 (3)	C9—C10—C11—C12	−176.83 (15)
C1—C2—C3—C8	1.51 (16)	C10—C11—C12—C13	0.1 (3)
C9—C2—C3—C8	−177.41 (13)	C11—C12—C13—C14	−1.4 (3)
C8—C3—C4—C5	2.3 (2)	C12—C13—C14—C15	1.4 (3)
C2—C3—C4—C5	178.11 (15)	C11—C10—C15—C14	−1.1 (2)
C3—C4—C5—C6	0.8 (2)	C9—C10—C15—C14	176.88 (14)
C3—C4—C5—Cl1	179.88 (11)	C13—C14—C15—C10	−0.1 (2)
C4—C5—C6—C7	−2.7 (2)	C2—C9—C16—C17	−179.56 (14)
Cl1—C5—C6—C7	178.18 (12)	C10—C9—C16—C17	1.9 (2)
C5—C6—C7—C8	1.4 (2)	C9—C16—C17—C22	168.54 (15)
C6—C7—C8—N1	−177.70 (16)	C9—C16—C17—C18	−11.6 (3)
C6—C7—C8—C3	1.8 (2)	C22—C17—C18—C19	−1.7 (2)
C4—C3—C8—N1	175.92 (12)	C16—C17—C18—C19	178.39 (14)
C2—C3—C8—N1	−0.98 (16)	C17—C18—C19—C20	0.8 (3)
C4—C3—C8—C7	−3.7 (2)	C18—C19—C20—C21	0.5 (3)
C2—C3—C8—C7	179.42 (14)	C19—C20—C21—C22	−0.7 (2)
C1—C2—C9—C16	−150.83 (15)	C20—C21—C22—C17	−0.4 (2)
C3—C2—C9—C16	27.9 (2)	C18—C17—C22—C21	1.5 (2)
C1—C2—C9—C10	27.8 (2)	C16—C17—C22—C21	−178.55 (14)
C3—C2—C9—C10	−153.48 (13)	C2—C1—N1—C8	0.95 (18)
C16—C9—C10—C11	94.92 (18)	C7—C8—N1—C1	179.63 (16)
C2—C9—C10—C11	−83.68 (17)	C3—C8—N1—C1	0.06 (18)

### Hydrogen-bond geometry (Å, °)

**Table:**

Cg1and Cg2 are the centroids of the C10–C15 and C3–C8 rings, respectively.

**Table:**

*D*—H···*A*	*D*—H	H···*A*	*D*···*A*	*D*—H···*A*
C18—H18···Cg1	0.93	2.74	3.573 (2)	150
C20—H20···Cg2^i^	0.93	2.97	3.690 (2)	136

Symmetry codes:  (i) −*x*+1, *y*−1/2, −*z*+1/2.
